# Differential regulation of protein expression in response to polyunsaturated fatty acids in the liver of apoE-knockout mice and in HepG2 cells

**DOI:** 10.1186/s12929-015-0118-2

**Published:** 2015-02-07

**Authors:** Chun-Ying Huang, Wei-Ming Chen, Yeou-Guang Tsay, Shu-Chen Hsieh, Yun Lin, Wen-Jane Lee, Wayne Huey-Herng Sheu, An-Na Chiang

**Affiliations:** Institute of Biochemistry and Molecular Biology, National Yang-Ming University, No.155, Sec.2, Li-Nong Street, Taipei, 11221 Taiwan; Division of Gastroenterology and Hepatology, Department of Internal Medicine, Chang Gung Memorial Hospital, Chiayi, 61363 Taiwan; Institute of Food Science and Technology, National Taiwan University, Taipei, 10617 Taiwan; Department of Medical Research, Taichung Veterans General Hospital, Taichung, 40354 Taiwan; Graduate Institute of Clinical Medical Sciences, College of Medicine, Chang Gung University, Taoyuan, 33302 Taiwan

**Keywords:** Polyunsaturated fatty acids (PUFAs), Proteomics, Inflammation, C-reactive protein (CRP), Nuclear factor-κB (NF-κB) pathway

## Abstract

**Background:**

Polyunsaturated fatty acids (PUFAs) are nutrients necessary for life. The liver is the essential metabolic center, which aids in maintaining health via diverse biological actions. In the present work, a proteomics study was conducted with an aim to provide new insights into PUFA-regulated hepatic protein expression in apoE-knockout mice. Additionally, we investigated how n-3 PUFAs influence cytokine-challenge by using HepG2 cells as a model.

**Results:**

Through the proteomic analysis using 2-dimensional electrophoresis and mass spectrometry, we found that 28, 23, 14, and 28 hepatic proteins were up-regulated at least a two-fold difference in intensity compared with the control group in mice treated with the docosahexaenoic acid, eicosapentaenoic acid, arachidonic acid, and linoleic acid, respectively. In contrast, 12 hepatic proteins were down-regulated with a ratio value of less than 0.5 compared to their control counterparts by these four fatty acids. All of the altered proteins were then sorted according to their biochemical properties related to metabolism, redox stress/inflammation, enzymatic reactions, and miscellaneous functions. The results provide evidence that PUFAs may act as either pro-inflammatory or anti-inflammatory agents. Cytokine-challenged HepG2 cells were used to reveal the anti-inflammatory function of n-3 PUFAs. The results showed that interleukin (IL)-1β combined with IL-6 induced C-reactive protein (CRP) mRNA expression and its protein secretion by HepG2 cells. The CRP promoter activity was significantly increased in the IL-6-treated cells, whereas IL-1β alone had no effect. However, IL-1β and IL-6 acted synergistically to further enhance CRP promoter activities. Furthermore, n-3 PUFAs inhibited nuclear factor-κB (NF-κB) activation and the phosphorylation of the nuclear signal transducer and activator of transcription 3 (STAT3) during cytokine-induced CRP production.

**Conclusion:**

This study indicates that PUFAs induced changes in the hepatic protein profile *in vivo*. Furthermore, n-3 PUFAs exert their anti-inflammatory properties through differential molecular mechanisms in hepatic cells. These results provide novel information regarding the roles of PUFAs in the liver at the tissue and cellular levels.

## Background

Dietary n-3 and n-6 polyunsaturated fatty acids (PUFAs) have multiple physiological functions in humans [1,2]. Ample evidence demonstrated that supplementation with n-3 PUFAs is able to decrease plasma triacylglycerol concentration, reduce inflammatory stress, and attenuate atherosclerotic progression [3-6]. Atherosclerosis is a cardiometabolic disease and the liver is an essential metabolic center in animals and humans. However, responses in terms of hepatic protein expression in animals or hepatocytes that have been challenged with different PUFAs are still poorly understood. The elucidation of the regulatory mechanisms involved in these processes will undoubtedly improve our understanding for dietary intervention when facing metabolic disorders, inflammation, obesity, carcinogenesis, and coronary artery diseases.

Previous studies have shown that n-3 PUFAs have a protective role in the prevention of cardiovascular disease [5-7]. Atherosclerosis is a cardiovascular disease associated with disorder of inflammatory signaling and lipid metabolism [8,9]. The liver is directly involved in the development of the cardiometabolic syndrome, which links metabolic disorder with cardiovascular complications [10]. Establishment of an early detection approach that can slow down or even stop atherosclerotic progression is of great interest. In the current study, we used apoE-knockout (apoE-KO) mice as an animal model to monitor hepatic protein expression in response to different PUFAs as a first step using the proteomics approach. This observation should provide important insights into the regulation of protein expression in the liver by PUFAs.

Consumption of n-3 PUFAs have been found to suppress inflammatory processes, which makes these fatty acids good candidates for both the prevention and amelioration of several organ-specific and systemic diseases [11,12]. Inflammation is usually characterized by cytokine activation. Furthermore, IL-1β and IL-6, either alone or in combination, influence hepatocyte protein synthesis and secretion [13,14]. Inflammatory stress has been suggested to act as a trigger for a complex series of molecular and biochemical changes that bring hepatic damages [15]. C-reactive protein (CRP) is a well-known risk marker for chronic inflammation and this protein is predominantly produced by hepatocytes [16]. We hypothesized that inflammatory stress-induced CRP secretion by the liver may be influenced by PUFAs. Herein, we tested the hypothesis that PUFAs may play a role in modulating the expression and secretion of CRP via different signaling pathways in cytokine-stimulated HepG2 cells, which has been shown with more similarity to human liver than the other cell lines in comparisons of the protein expression [17].

The aim of this study was to investigate the roles of PUFAs in the regulation of hepatic protein expression, *in vivo,* and in the regulation of the signaling pathway involved in cytokine-induced CRP expression, *in vitro*. First, we elucidated the changes in hepatic protein expression in apoE-KO mice that had been treated with the n-3 PUFAs, docosahexaenoic acid (22:6; DHA) and eicosapentaenoic acid (20:5; EPA), and the n-6 PUFAs, arachidonic acid (20:4; AA) and linoleic acid (18:2; LA), by using a proteomic approach. We then investigated how PUFAs regulate cytokine-induced CRP expression in HepG2 cells. Signaling events and CRP gene regulation that occur in IL-β and IL-6-challenged cells were also investigated. Finally, we propose a model that aims to describe the mechanisms by which n-3 PUFAs regulate hepatic signaling pathway and CRP gene expression.

## Methods

### Animals

ApoE-KO mice were originally obtained from Jackson laboratory (Bar Harbor, ME, USA). Forty 12-week-old-male mice were bred at the animal center of National Yang-Ming University. The study followed the Guide for the Care and Use of Laboratory Animals published by the US National Institutes of Health (NIH Publication No. 85–23, revised 1996) and all experimental procedures were approved by the Animal Care and Utilization Committee of National Yang-Ming University, Taipei, Taiwan. After one week on a commercial mouse chow diet, the mice were randomly allocated to one of five groups (n = 8). The control group was given normal laboratory mouse diet *ad libitum* and 1.1% ethanol in PBS (150 mM NaCl, 20 mM sodium phosphate, pH 7.4) by gavage every day. The other four groups were fed the same normal diet *ad libitum* plus 200 mg/kg of DHA, EPA, AA, or LA in 1.1% ethanol/PBS every day by gavage. After 10 weeks on the diet, the mice were fasted overnight, and their body weight was recorded. The mice were then euthanized and blood and liver samples were collected at the end of the experiment. The blood was centrifuged at 12000 *g* for 15 min and the plasma supernatant was then stored at −35°C until analysis. Liver tissues were harvested, washed with ice-cold isotonic saline, and stored at −80°C until use.

### Determination of plasma and hepatic lipid levels

Plasma derived from apoE-KO mice was diluted with 150 mM NaCl, 1 mM EDTA (pH 7.4) so that the OD measurement and lipid concentrations were brought into the normal range. The plasma triacylglycerol (TG), total cholesterol (TC), HDL-cholesterol, and LDL-cholesterol concentrations were assayed enzymatically using commercial kits (Wako Chemicals, Richmond, VA, USA). Lipids were extracted from liver samples following the modified method described by Folch et al. [18]. Briefly, total lipids were extracted from the liver samples by homogenizing the tissues with 8:4:3 chloroform/methanol/0.9% NaCl (v/v) to a final dilution of 20-fold the original volume of the tissue sample. The organic layer was then separated, evaporated, and reconstituted in chloroform. The values of TG and TC were normalized against the weight of the extracted liver.

### Proteome analysis

Mouse liver tissue was cut into small pieces and ground in sample buffer (40 mM Tris–HCl, pH 7.6, 7 M urea, 2 M thiourea, 4% CHAPS, 10 mM 1,4-dithioerythritol, 1 mM EDTA) in the presence of a mixture of protease inhibitors (1 mM phenylmethanesulfonyl fluoride (PMSF) and 1 μg/mL of each pepstatin A, chymostatin, leupeptin, and antipain). Two-dimensional (2-D) gel electrophoresis was performed as in a recent proteomics study [19]. Briefly, isoelectric focusing (IEF) was carried out with IPG strip gels (Bio-Rad Laboratories) according to the manufacturer’s instructions at 20 V for 3 h, 500 V for 3 h, 1000 V for 3 h, 4000 V for 3 h, 6000 V for 3 h, and finally at 8000 V for more than 3 h using an IPGphor IEF system (Amersham Pharmacia Biotech, Piscataway, NJ, USA). After IEF, the immobilized pH gradient strips were equilibrated for 15 min in equilibration buffer [50 mM Tri-HCl, pH 8.8, 6 M urea, 30% glycerol, 2% SDS, 2% dithiothreitol (DTT), and 0.002% bromophenol blue], which was followed by another 15 min incubation in the same buffer containing 2.5% iodoacetamide in place of DTT. Next, 2-D SDS-polyacrylamide gel electrophoresis (SDS-PAGE) was performed on a 12.5% polyacrylamide gel using a Protein II xi 2-D cell System (Bio-Rad Laboratories) at 35 mA and 20°C for 6 h. After electrophoresis, the gel was stained with Coomassie blue G-250 (Bio-Rad Laboratories) for 16 h and then destained with distilled water until the background turned clear. The gel was then analyzed using an ImageMaster 2D-Elite (Amersham Pharmacia Biotech) for spot detection, quantification, and matching. Differentially expressed spots were analyzed and annotated. The tryptic peptides produced by the protease digestion were extracted from the gel plugs by treatment with 0.1% formic acid in 50% ACN. Electrospray ionization tandem mass spectrometry was performed on the extracted peptides using a ThermoFinnigan LCQ Deca ion trap mass spectrometer (Thermo Electron, Mountain View, CA, USA) coupled with an Agilent 1100 HPLC system (Agilent Technologies, Inc., Santa Clara, CA, USA). The acquired collision induced dissociation spectra were interpreted using TurboSequest software (ThermoFinnigan), which matches tandem mass spectra against a non-redundant protein database.

### Cell culture

The human hepatoma cell line HepG2 was cultured in Dulbecco’s modified Eagle’s medium (DMEM) supplemented with 10% (v/v) heat-inactivated fetal bovine serum (FBS), 100 units/mL penicillin, 100 mg/mL streptomycin, 0.3 mg/mL L-glutamine and 0.1 mM nonessential amino acids at 37°C in 5% CO_2_. The cell culture media and the fatty acid free FBS were purchased from GIBCO (Carlsbad, CA, USA). The final concentrations of 80, 100, and 120 μM of each PUFA were diluted by the cell culture media from 40 mM PUFA stock solution. The fatty acids media were mixed with fatty acid-free bovine serum albumin (BSA) stock solution in cell culture media. The final concentration of BSA was adjusted to 0.012% (w/v) in each PUFA group. After treatment with PUFAs and cytokines, the cell medium was centrifuged at 3,000 *g* for 5 min and the supernatant was collected in order to determine CRP secretion. The cytosolic protein was prepared by incubating cells in lysis buffer (10 mM Tris, pH 7.4/0.32 M sucrose/2 mM 2-mercaptoethanol/1% Triton X-100/1X protease inhibitor cocktail). The cell lysates were separated by centrifugation at 12000 *g* for 20 min at 4°C and stored at −80°C until use.

### Cell viability assessment

The 3-(4,5-dimethylthiazol-2-yl)-2,5-diphenyltetrazolium bromide (MTT) assay was employed to determine the effect of PUFAs at 80, 100, and 120 μM on cell viability. Following treatment with PUFAs for 24 h, the cells were stimulated with IL-1β and IL-6 (10 ng/mL) for an additional 24 h and then incubated with MTT (0.5% in PBS) for 4 h at 37°C. The purple formazan crystals formed were then dissolved in 0.002% Triton X-100/1% HCl-isopropanol. The extent of MTT reduction was quantified using a microplate reader at 550 nm and 690 nm. The viability of the samples is expressed relative to that of control cells (100%).

### RT-PCR and real-time quantitative PCR

Total RNA from 1 × 10^6^ cells was extracted with TRIzol reagent (Invitrogen, Carlsbad, CA, USA). A sample of 1 μg purified RNA was treated with RNase-free DNAse I (Invitrogen), and then reverse-transcribed with oligo(dT) primer using the SuperScript First-Strand Synthesis System (Invitrogen) in order to generate cDNA. The PCR was performed in the buffer containing 10 mM Tris/HCl, pH 8.3, 50 mM KCl, 2.5 mM MgCl_2_, 0.2 μM each dNTP, 1 μM each primer, and 1 unit of ExTAQ DNA polymerase, and put on a Peltier Thermal Cycler (MJ Research, Model PTC-200). The sequences of the PCR primers for the *CRP* gene amplification were as follows: forward, 5’-CCTATGTATCCCTCAAAGCA-3’ and reverse, 5’-CCCACAGTGTATCCCTCTT-3’. The porphobilinogen deaminase (*PBGD*) gene was used as an internal control and its primer sequences were as follows: forward 5’-AGGATGGGCAACTGTACC-3’ and reverse 5’-GTTTTGGCTCCTTTGCTCAG-3’. The relative mRNA levels of *CRP* were normalized against the level of *PBGD* mRNA expression in each assay. Real-time quantitative PCR was performed with SYBR green master mixture (Qiagen, Valencia, CA, USA) in a LightCycler Carousel-Based System (Roche). The primers were used as follows: CRP: forward, 5’-ACTTCCTATGTATCCCTCAAAG-3’ and reverse, 5’-CTCATTGTCTTGTCTCTTGGT-3’. Glyceraldehyde-3-phosphate dehydrogenase (GAPDH): forward, 5’-GAAGGTGAAGGTCGGAGTC-3’ and reverse, 5’-GAAGATGGTGATGGGATTTC-3’. Quantification of CRP mRNA was calculated by the Ct method (ratio = 2 - (Ct(CRP) - Ct(GAPDH))) as described previously by Patel et al. [20].

### Western blot analysis

The effect of PUFAs on the secretion of CRP into the cell medium and the STAT3 expression in total cell lysate was determined by western blot analysis. The nuclear fraction was collected to examine the effects of PUFAs on IL-6-induced nuclear translocation of STAT3 and NF-κB subunits p65 and p50. For nuclear protein isolation, cell lysates were incubated in extraction buffer containing 20 mM HEPES, pH 7.4, 10 mM KCl, 1 mM MgCl_2_, 0.5% Nonidet P40, 0.5 mM DTT and complete protease inhibitor cocktail (Roche, Hercules, CA, USA). After centrifugation at 3000 *g* for 10 min, the pellet was resuspended in cold nuclear extraction buffer [20 mM HEPES, pH 7.4, 0.4 M NaCl, 10 mM KCl, 1 mM MgCl_2_, 0.5 mM DTT and 20% (v/v) glycerol supplemented with complete protease inhibitor cocktail (Roche)]. The protein concentrations of the cell medium, cell lysates, and nuclear fractions were determined using the Bradford assay (Bio-Rad). Equal amounts of proteins were subjected to 10% SDS-PAGE and then transferred onto polyvinylidene difluoride membrane (Millipore, Billerica, MA, USA) after gel electrophoresis. The blots were blocked with 5% (w/v) skim milk and probed with a primary antibody and then immunoblotted using a specific horseradish peroxidase-conjugated secondary antibody. The blots were stripped for further probing with albumin, α-tubulin, or B23 antibodies as the internal controls for medium protein, cytosolic proteins, or nuclear proteins, respectively. Bound IgG was visualized using an enhanced chemiluminescence detection kit system (PerkinElmer, Shelton, CT, USA), and quantified by ImageQuant 5.2 software (Healthcare Bio-Sciences, Pennsylvania, USA).

### Transient transfection and reporter assays

Transient transfection was performed using Lipofectamine 2000 reagents (Gibco BRL, Grand Island, NY, USA) according to the manufacturer’s instructions. The human *CRP* promoter fragment from −300/+1 was amplified using the primers 5’-CCGACGCGTACCCAGATGGCCACTCGTTTAATATGTTACC-3’ and 5’-CCTAGATCTAGAGCTACCTCCTCCTGCCTGG-3’, which contain *Mlu*I and *Bgl*II restriction sites. The PCR products were cloned into the luciferase reporter pGL3 basic vector (Promega), and the DNA sequence was verified by sequencing of the clones. HepG2 cells were cotransfected with 1 μg of CRP promoter-driven luciferase reporter plasmid and 0.5 μg of pCMV-β-galactosidase expression plasmid in serum-free DMEM. After 24 h of transfection, the cells were pretreated with 100 μM PUFAs for 24 h before stimulation with 10 ng/mL of IL-1β and/or IL-6 for an additional 12 h. Next, the cells were lysed with lysis buffer (70 mM K_2_HPO_4_, 2.1 mM MgCl_2_, 55 mM Tris–HCl, pH 7.8, 0.7 mM DTT and 1% Triton X-100) and the cell lysates were harvested by centrifugation. β-galactosidase activity was measured by incubating the cell extracts with *o*-nitrophenyl-β-D-galactopyranoside (Sigma-Aldrich, St. Louis, MO, USA). Relative luciferase activity is presented as firefly luciferase values normalized against the β-galactosidase activity.

### Statistical analysis

Data are expressed as mean ± SEM of at least three independent experiments. Comparisons between control and experimental groups were made using a one-way ANOVA with the Tukey’s method as a *post hoc* test. *P* < 0.05 was considered statistically significant.

## Results

### Plasma and hepatic lipid levels in response to PUFA treatment

The mice used in this study remained healthy and were similar in appearance. No significant difference was observed between the PUFA and control groups for most of the biochemical parameters (Table 1). However, plasma levels of triacylglycerol were significantly decreased in the DHA-fed and EPA-fed mice (1.1 ± 0.1 and 1.1 ± 0.1 mM, respectively) compared to those of the control mice (1.5 ± 0.1 mM). In contrast, plasma total cholesterol levels were significantly higher in the LA-fed group, while plasma levels of HDL-cholesterol and LDL-cholesterol were not statistically different among the five groups. Moreover, hepatic levels of triacylglycerol and cholesterol in PUFA-treated mice were also found not to be significantly different to those of control mice.

**Table 1:** **Biometric parameters of the control and the experimental groups**

	**Control**	**DHA**	**EPA**	**AA**	**LA**
BW (g)	26.2 ± 2.8	25.5 ± 2.2	25.9 ± 2.8	26.4 ± 2.1	27.4 ± 2.3
TG (mM)	1.5 ± 0.1	1.1 ± 0.1*	1.1 ± 0.1*	1.4 ± 0.3	1.6 ± 0.3
TC (mM)	22 ± 1.8	22.6 ± 1.7	22.4 ± 1.1	23.3 ± 0.9	24.3 ± 1.4*
HDL-C (mM)	2.5 ± 0.4	2.6 ± 0.4	2.5 ± 0.5	2.7 ± 0.5	3 ± 0.5
LDL-C (mM)	4.2 ± 1.3	4.4 ± 0.9	4.6 ± 1.2	4.8 ± 1.3	4.9 ± 1.2
LW (g)	1.2 ± 0.2	1.3 ± 0.2	1.2 ± 0.1	1.2 ± 0.1	1.3 ± 0.1
HTG (mg/g liver)	95.5 ± 22.2	93.7 ± 14.3	90.5 ± 19.5	97.1 ± 17.6	106.6 ± 17.9
HTC (mg/g liver)	46.1 ± 9.6	42.3 ± 9.8	43.9 ± 11.7	51.2 ± 11.2	48.1 ± 8.9

CN, control; DHA, docosahexaenoic acid; EPA, eicosapentaenoic acid; AA, arachidonic acid; LA, linoleic acid; BW, body weight; TG, triacylglycerol; TC, total cholesterol; HDL-C, high-density lipoprotein-cholesterol; LDL-C, low-density lipoprotein-cholesterol; LW, liver weight; HTG, hepatic triacylglycerol; HTC, hepatic total cholesterol.

*P <0.05 vs. control.

### Expression of hepatic proteins in the PUFA-treated apoE-KO mice

**Figure 1:**
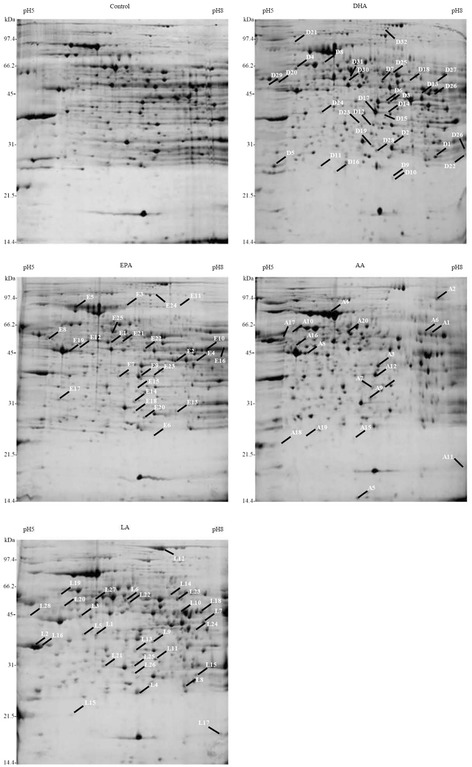
**Representative 2-D gel map of the hepatic proteins in apoE-KO mice.** Mice were fed a chow diet (control) *ad libitum* and animals in the experimental groups were fed with chow diet plus 200 mg/kg of docosahexaenoic acid (DHA), eicosapentaenoic acid (EPA), arachidonic acid (AA), or linoleic acid (LA) in 1.1% ethanol/PBS every day by gavage. The proteins extracted from the liver were separated initially using a pH 5–8 IPG strip as the first dimension and then on a 12.5% SDS-polyacrylamide gel as the second dimension. The gels were stained with Coomassie blue G-250 and molecular weight standards are shown on the left. The differentially modulated protein spots are numbered and their identities are reported in Table 2.

A proteomics study was conducted to identify PUFA-regulated proteins in the liver of apoE-KO mice. Changes in protein levels were determined by comparison of the intensities of the corresponding spots on 2-D gels. Figure 1 shows a representative example of the hepatic proteins separated by 2-D gel electrophoresis. Proteins with pIs between 5 and 8 and molecular weights ranging from 14 kDa to 165 kDa were resolved. An average of about 700–1100 spots/gel was detected in the area included in the analysis using the 2-D ImageMaster software on the Coomassie blue-stained gel. Protein spots were selected if spot intensities were more than 2-fold increased or had a ratio value of less than 0.5 compared to their control counterparts. We utilized at least four replicate 2-D gels to confirm the presence of significant differences in spot intensities between the control and the PUFA-treated mice. The protein identity in the selected spots was then determined using liquid chromatography-tandem mass spectrometric analysis. The relative ratios of the identified proteins are listed in Table 2. Ninety-three proteins were significantly up-regulated and 12 proteins were remarkably down-regulated by the four PUFAs. These differentially expressed proteins have been divided into four categories according to their major biochemical functions, namely, metabolism, redox stress/inflammation, enzyme, and other miscellaneous functions. Table 2 gives a summary of the differentially expressed proteins for each experimental group compared to the control group. We found that 28, 23, 14, and 28 proteins were up-regulated in mice treated with DHA, EPA, AA, and LA, respectively. ApoE-KO mice treated with DHA, EPA, and LA presented more up-regulated hepatic proteins. In contrast, 4, 2, 6, and 0 proteins were down-regulated in the DHA, EPA, AA, and LA groups, respectively. Of the 93 proteins that were up-regulated in response to PUFA feeding, up to 29 proteins are known to be associated with redox stress and inflammation. Interestingly, 31 of the 105 PUFA-regulated proteins are thought to be NF-κB-responsive. However, their impact and physiological significance still need to be evaluated.

**Table 2:** **Summary of the regulated proteins present in the liver of apoE KO mice treated with different PUFAs**

**Spot no.** ^**(a)**^	**Protein** ^**(b)**^	**Ratio PUFA/control** ^**(c)**^	**Accession number** ^**(b)**^
	**Metabolism**		
D13	Fumarylacetoacetase	3.3	gi|544273
D14	Fructose-1,6-bisphosphatase 1	2.9	gi|14547989
D17	Malate dehydrogenase	2.6	gi|126889
D20	Oxysterols receptor LXR alpha	2.3	gi|12644486
D25	Aldehyde dehydrogenase	2.1	gi|1352250
D26	Isocitrate dehydrogenase	2.1	gi|6647554
D31	SREBP-1	0.5	gi|7144550
D32	Pyruvate carboxylase	0.5	gi|6679237
E8	ATP synthase beta chain	3.1	gi|20455479
E9	Malate dehydrogenase	2.8	gi|126889
E10	Isocitrate dehydrogenase	2.6	gi|6647554
E22	Alpha enolase	2.0	gi|13637776
E23	Fructose-1,6-bisphosphatase 1	2.0	gi|14547989
E24	Pyruvate carboxylase	0.5	gi|6679237
E25	SREBP-1	0.5	gi|7144550
A10	Pyruvate kinase isozyme M2	2.6	gi|2506796
A12	Malate dehydrogenase	2.2	gi|126889
A13	Glycerol-3-phosphate dehydrogenase	2.1	gi|121557
A14	Ketohexokinase	2.1	gi|6016435
L3	Succinyl-CoA synthetase	4.8	gi|52788305
L9	Fructose-1,6-bisphosphatase 1	3.4	gi|14547989
L10	Fumarylacetoacetase	3.2	gi|544273
L14	Phosphoglucomutase	2.8	gi|21362784
L27	Vitamin D-binding protein precursor	2.0	gi|46397761
	**Redox stress/Inflammation**		
D1	Annexin A2	8.2	gi|13435564
D6	Interleukin 1 receptor accessory	4.2	gi|19882203
D9	Peroxiredoxin 6	3.7	gi|6671549
D10	GlutathioneS-transferase, pi1	3.5	gi|10092608
D15	MAP kinase kinase 3	2.9	gi|1771303
D21	A-kinase anchor protein	2.3	gi|2852699
D22	Glutathione S-transferase Mu 5	2.3	gi|1346207
D23	Electron transfer flavoprotein alpha-subunit	2.2	gi|21759113
D24	DC-SIGN related protein 1	2.2	gi|46395849
D30	Selenium bind protein 2	0.5	gi|9507079
E1	Selenium bind protein 2	6.9	gi|9507079
E6	Peroxiredoxin 6	3.6	gi|3219774
E7	G-protein coupled receptor	3.1	gi|460318
E12	Interleukin 6 receptor, alpha	2.5	gi|7110655
E13	Glutathione S-transferase theta 2	2.4	gi|81916034
E16	Glucocorticoid-attenuated response gene 49 protein	2.2	gi|6831574
E18	Peroxiredoxin 4	2.1	gi|3024715
A5	Superoxide dismutase 1, soluble	4.7	gi|45597447
A11	Cyclophilin A	2.3	gi|118105
A20	Selenium bind protein 2	0.5	gi|9507079
L4	Glutathione peroxidase 1	4.7	gi|84871986
L6	Interferon regulatory factor	3.6	gi|972949
L7	Adrenoleukodystrophy protein	3.6	gi|6651050
L8	Glutathione S-transferase Mu 1	3.4	gi|121716
L15	Glutathione transferase omega-1	2.7	gi|6016174
L19	Heat shock protein 60	2.4	gi|51702252
L20	Mitogen-activated protein kinase 10	2.3	gi|2499604
L23	Farnesoid X-activated receptor	2.2	gi|21263825
L25	Peroxiredoxin 4	2.1	gi|3024715
	**Enzyme**		
D2	Glycine N-methyltransferase	7.1	gi|6754026
D5	DEAD-box RNA helicase	4.3	gi|6014946
D7	Cyp2c70 protein	3.8	gi|19387996
D8	3’-phosphoadenosine-5’-phosphosulfate synthase 1	3.7	gi|6754982
D16	Isopentenyl-diphosphate delta-isomerase 1	2.7	gi|13878548
D19	Indolethylamine N-methyltransferase	2.4	gi|731019
D27	Homogentisate 1,2-oxygenase	2.1	gi|7387755
D28	Thiopurine S-methyltransferase	2.0	gi|6094505
E2	Dehydrodolichyl diphosphate synthase	5.9	gi|46395956
E20	Thiopurine S-methyltransferase	2.1	gi|6094505
A17	Ubiquitin carboxyl-terminal hydrolase L1	0.4	gi|20178168
A19	Hypoxanthine guanine phosphoribosyl transferase 1	0.5	gi|13435621
L2	Amine N-sulfotransferase	5.1	gi|81870419
L11	3-hydroxyanthranilate 3,4-dioxygenase	2.9	gi|3929397
L12	Caramoyl-phosphate synthase I	2.8	gi|117492
L17	Era-like-GTPase	2.5	gi|19852066
L18	Betaine-homocysteine S-methyltransferase	2.5	gi|5915784
L26	Thiopurine S-methyltransferase	2.0	gi|6094505
	**Miscellaneous**		
D3	Hypothetical protein LOC74919	6.8	gi|33563309
D4	Chaperonin groEL precursor	5.4	gi|72957
D11	EF hand domain	3.4	gi|13386360
D12	Growth-arrest-specific protein 2	3.4	gi|120945
D18	Glial fibrillary acidic protein	2.6	gi|417050
D29	Protein disulfide-isomerase A6 precursor	0.4	gi|2501206
E3	RB-associated-KRAB repressor	5.1	gi|29789126
E4	Hypothetical protein DKFZp547P193.1	4.3	gi|11346331
E5	Myc-binding protein-associated protein	3.7	gi|24762234
E11	SEC63	2.6	gi|31981948
E14	START domain	2.4	gi|9910482
E15	Growth-arrest-specific protein 2	2.2	gi|120945
E17	Alpha-soluble NSF attachment	2.2	gi|17380315
E19	Beta-actin	2.1	gi|46397316
E21	Selenium bind protein 1	2.1	gi|22164798
A1	Procollagen, type XXIII, alpha 1	9.8	gi|23510253
A2	Advillin	8.3	gi|6857753
A3	Putative TDPOZ1 protein	7.7	gi|38077962
A4	Serum albumin precursor	7.2	gi|5915682
A6	Hypothetical protein LOC225913	4.5	gi|21703976
A7	Growth-arrest-specific protein 2	3.4	gi|120945
A8	Beta-actin	3.1	gi|46397316
A9	START domain	2.8	gi|9910482
A15	Chimerin 1 isoform 2	0.4	gi|13386436
A16	Rab GDP dissociation inhibitor beta	0.4	gi|1707891
A18	Rho GDP dissociation inhibitor 1	0.4	gi|21759130
L1	Upp2 protein	5.3	gi|20071298
L5	Eukaryotic translation initiation factor 3 subunit 2	3.9	gi|20138778
L13	Growth-arrest-specific protein 2	2.8	gi|120945
L16	Regucalcin	2.6	gi|2498920
L21	Caspase-6 precursor	2.3	gi|2493529
L22	Guanosine diphosphate dissociation inhibitor 2	2.2	gi|13638229
L24	Transducin alpha-1 chain	2.1	gi|121033
L28	Apolipoprotein A-IV precursor	2.0	gi|1703331

(a) The spot numbers correspond to the same protein signal that was detected in the Figure 1.

(b) The protein names and accession numbers were taken from the NCBI database.

(c) Ratio of the protein intensity in each PUFA-treated group to the corresponding spot in the control group represents average values of triplicate samples.

### Effects of PUFAs on CRP protein secretion and *CRP* mRNA expression in HepG2 cells

**Figure 2:**
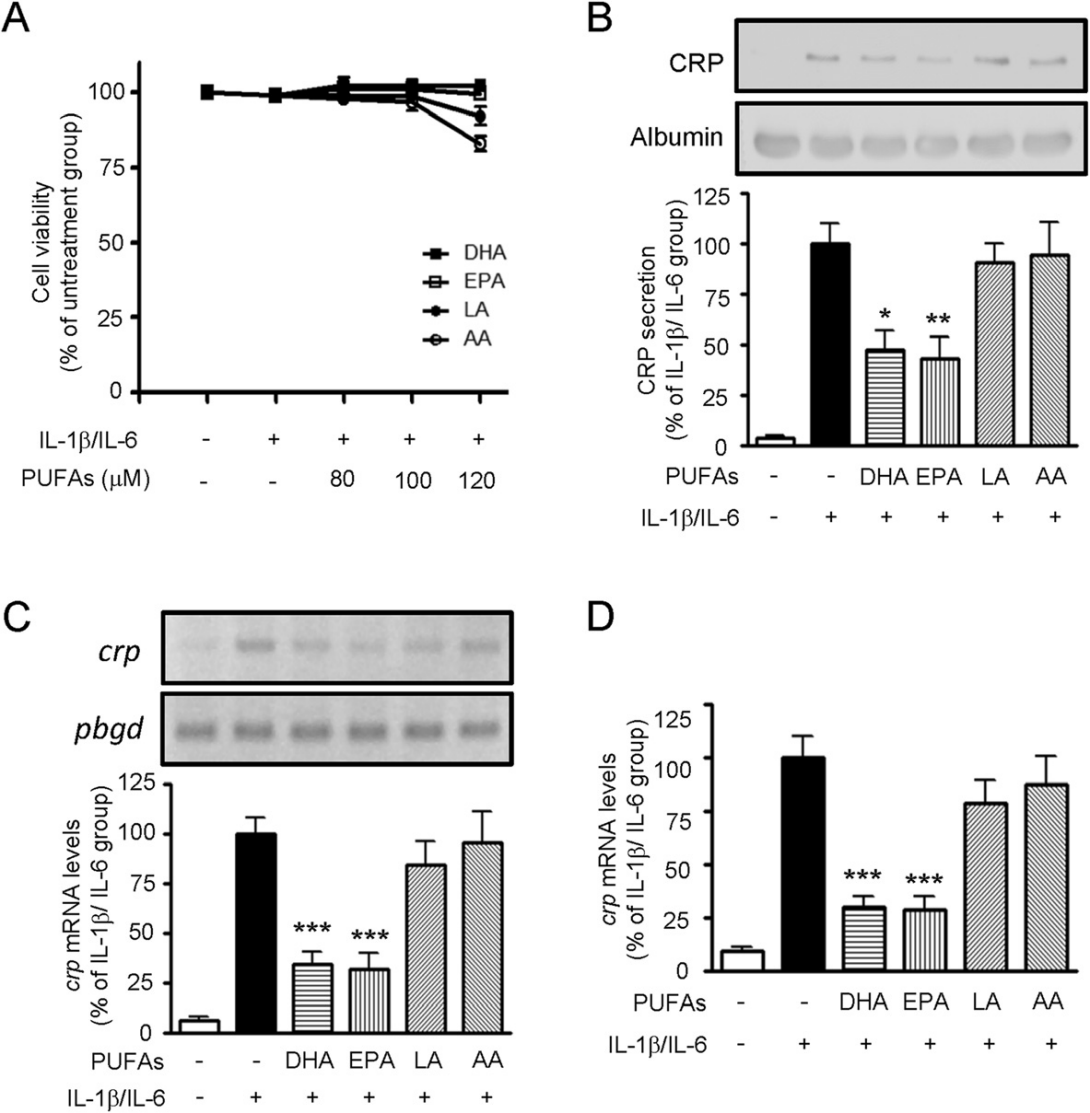
**Effects of PUFAs on cell viability, CRP secretion, and** ***CRP*** **mRNA expression in HepG2 cells. (A)** Cell viability was measured by MTT assay after 24 h of PUFA treatment and after a further 24 h of IL-1β/IL-6-stimulation. Cell viability is expressed as a percentage relative to the control value from cells without PUFA treatment and cytokine challenge. **(B)** Cells were treated with 10 ng/mL of IL-1β/IL-6 for 24 h followed by 100 μM PUFAs for another 24 h. The amount of CRP released into the cell medium was determined by western blot analysis. Pretreatment of HepG2 cells with DHA and EPA resulted in an inhibition of *CRP* mRNA expression as measured by RT-PCR **(C)** and real-time quantitative PCR **(D)**. Data are expressed as the mean ± SEM of at least three independent experiments, *P < 0.05, **P < 0.01, ***P < 0.001 compared to the IL-1β/IL-6-treated cells.

Taking into account the fact that a large number of the PUFA-regulated hepatic proteins were greatly associated with inflammation as shown in Table 2, the mechanisms of PUFAs on the regulation of inflammatory protein CRP expression were then elucidated using HepG2 cells. Initially, HepG2 cells were incubated with various concentrations (80, 100, and 120 μM) of PUFAs for 24 h and were stimulated with 10 ng/mL of IL-1β plus IL-6 for another 24 h. The effect of PUFAs and cytokine on cell viability was determined by MTT assay. No significant toxicity was observed among the cells stimulated by PUFAs below 120 μM and IL-1β plus IL-6 at the same time (Figure 2A). Considering that CRP is an inflammatory protein mainly produced by hepatocytes, the regulation of CRP secretion and *CRP* mRNA expression by PUFAs were then investigated. Figure 2B shows that DHA and EPA induced a 52.7% and 56.6% reduction in CRP secretion, respectively, when compared to the IL-1β and IL-6-stimulated control group. Furthermore, the IL-1β and IL-6 treatment caused a 15.6-fold increase in *CRP* mRNA expression and that DHA and EPA inhibited this induction by more than half (Figure 2C). In contrast, LA and AA did not have any effect on the regulation of CRP secretion. We also determined the effect of the PUFAs on *CRP* mRNA expression by real-time quantitative PCR. As shown in Figure 2D, IL-6-induced *CRP* mRNA expression was significantly inhibited by DHA and EPA, while treatment with LA and AA did not affect the *CRP* mRNA expression. All of these results pointed to the anti-inflammatory role of n-3 PUFAs in hepatocytes.

### Effects of PUFAs on CRP promoter activity

**Figure 3:**
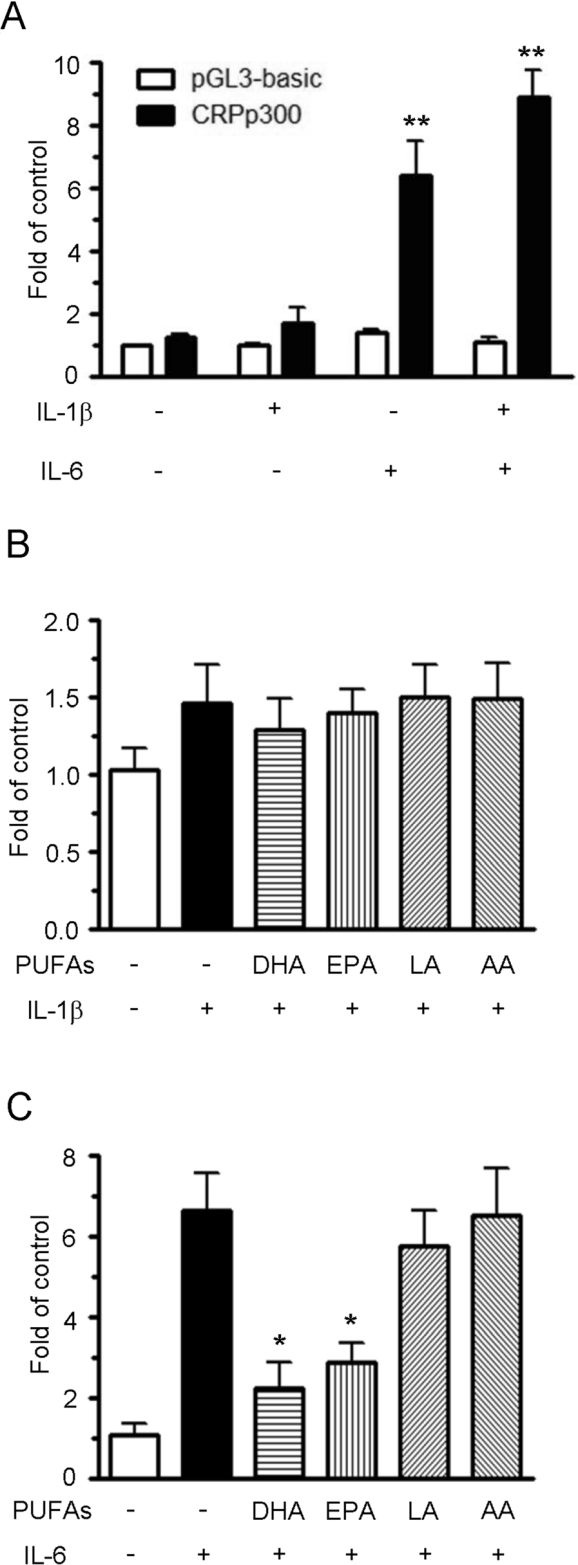
**Effects of IL-1β and IL-6 on** ***CRP*** **gene transcription.** HepG2 cells were co-transfected with a *CRP* promoter reporter construct and a β–galactosidase construct. After transfection for 24 h, the cells were treated with 10 ng/mL of IL-1β/IL-6 **(A)**, with10 ng/mL of IL-1β alone **(B)** or with 10 ng/mL of IL-6 alone **(C)** for an additional 12 h. The cell lysates were processed and the luciferase activity was normalized against β-galactosidase activity in order to correct for transfection efficiency. Relative luciferase units are indicated as the relative value of the activity of the untreated group transfected with the pGL3-basic vector (set as 1). Results are presented as the mean ± SEM of at least three independent experiments. *P < 0.05 compared to the IL-6-treated group, **P < 0.01 compared to the vehicle-treated group.

To examine whether the effect of cytokine-induced CRP gene expression is mediated through transcription regulation, studies were carried out on HepG2 cells transiently transfected with a CRP-luciferase construct that contained the CRP 5’-flanking region consisting of −300 bp to +1 bp. Under cytokine challenge, IL-6 induced CRP promoter activity by 4.6-fold, but no effect was observed when the cells were treated with IL-1β alone. However, a combination of IL-1β and IL-6 synergistically induced CRP promoter activity by 7.9-fold (Figure 3A). These results indicate that IL-6 appears to serve as the major mediator, causing the up-regulation of CRP gene expression. In addition, IL-1β acts cooperatively with IL-6 during the induction of CRP promoter activity. Since the findings revealed that IL-1β and IL-6 affected CRP promoter activity in different ways, we then investigated whether PUFAs might play different roles in CRP gene regulation in HepG2 cells challenged with different cytokines. Luciferase assays were carried out to explore this hypothesis and showed that CRP promoter activity was not affected by PUFAs in cells treated with IL-1β alone (Figure 3B), but that DHA and EPA reduced the IL-6-induced CRP promoter activity (Figure 3C). However, LA and AA had no significant effect on the regulation of CRP promoter activity in the IL-6-challenged cells.

### Regulation of nuclear p65 and p50 expression by DHA and EPA

**Figure 4:**
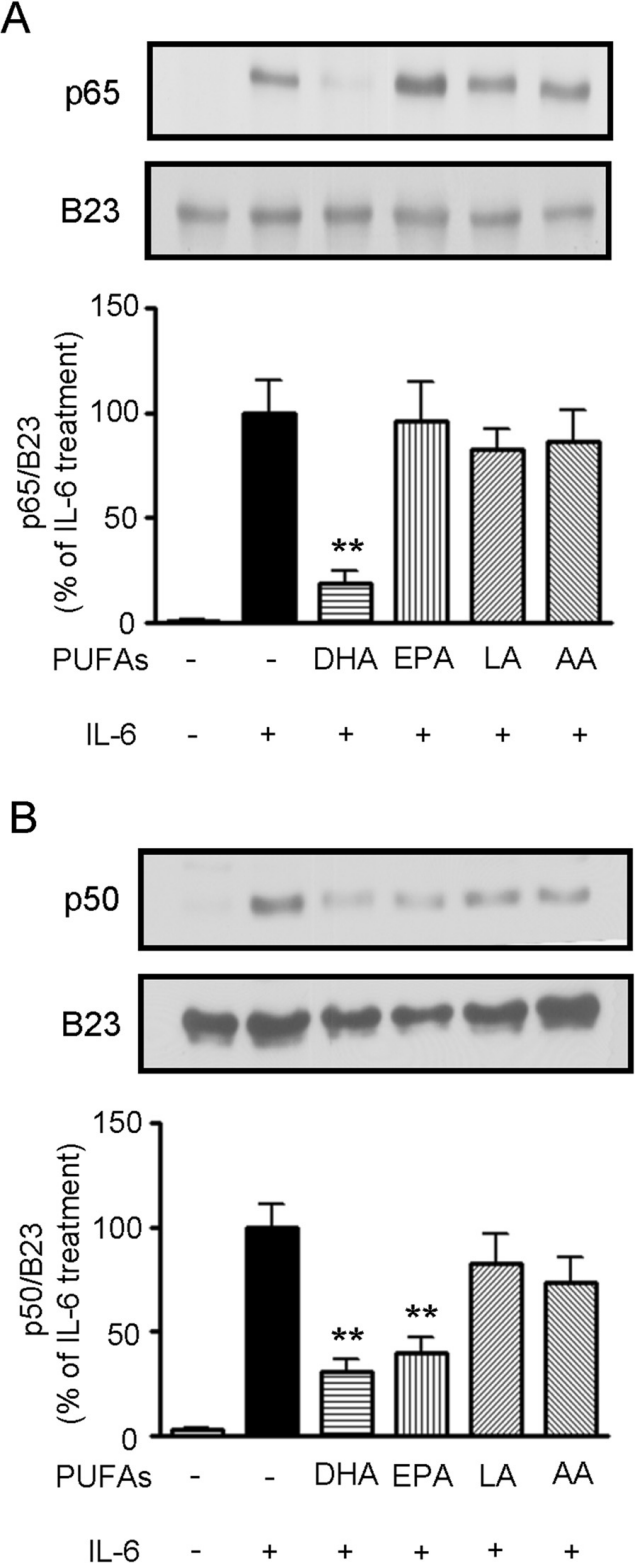
**Effects of PUFAs on IL-6-induced NF-κB subunit translocation to the nucleus in HepG2 cells.** Cells were pretreated with 100 μM PUFAs for 24 h before stimulation with 10 ng/mL of IL-6 for an additional 30 min. The levels of nuclear NF-κB subunits p65 **(A)** and p50 **(B)** were detected by western blot analysis. The expression of B23 was used as the internal control for the nuclear protein. Data were quantified by densitometry and the value of the IL-6-treated group was set as 100%. Results are presented as the mean ± SEM of at least three independent experiments, **P < 0.01 compared to the IL-6-treated group.

It has been reported that the NF-κB signaling pathway leads to a series of inflammatory reactions [21]. To examine whether down-regulation of IL-6-induced hepatic CRP expression by DHA and EPA occurs through the NF-κB signaling pathway, the effect of PUFAs on the regulation of nuclear p65 and p50 expression was measured by western blot analysis. As shown in Figure 4A, DHA remarkably suppressed nuclear NF-κB subunit p65 (also known as RelA) expression, while EPA, LA, and AA had no effect when compared to the IL-6-stimulated group. A similar assay was conducted to explore nuclear p50 expression. Both DHA and EPA were found to inhibit nuclear p50 expression in the IL-6-treated cells (Figure 4B). However, LA and AA did not have any effect on the regulation of nuclear p50 expression.

### Effects of PUFAs on STAT3 activation

**Figure 5:**
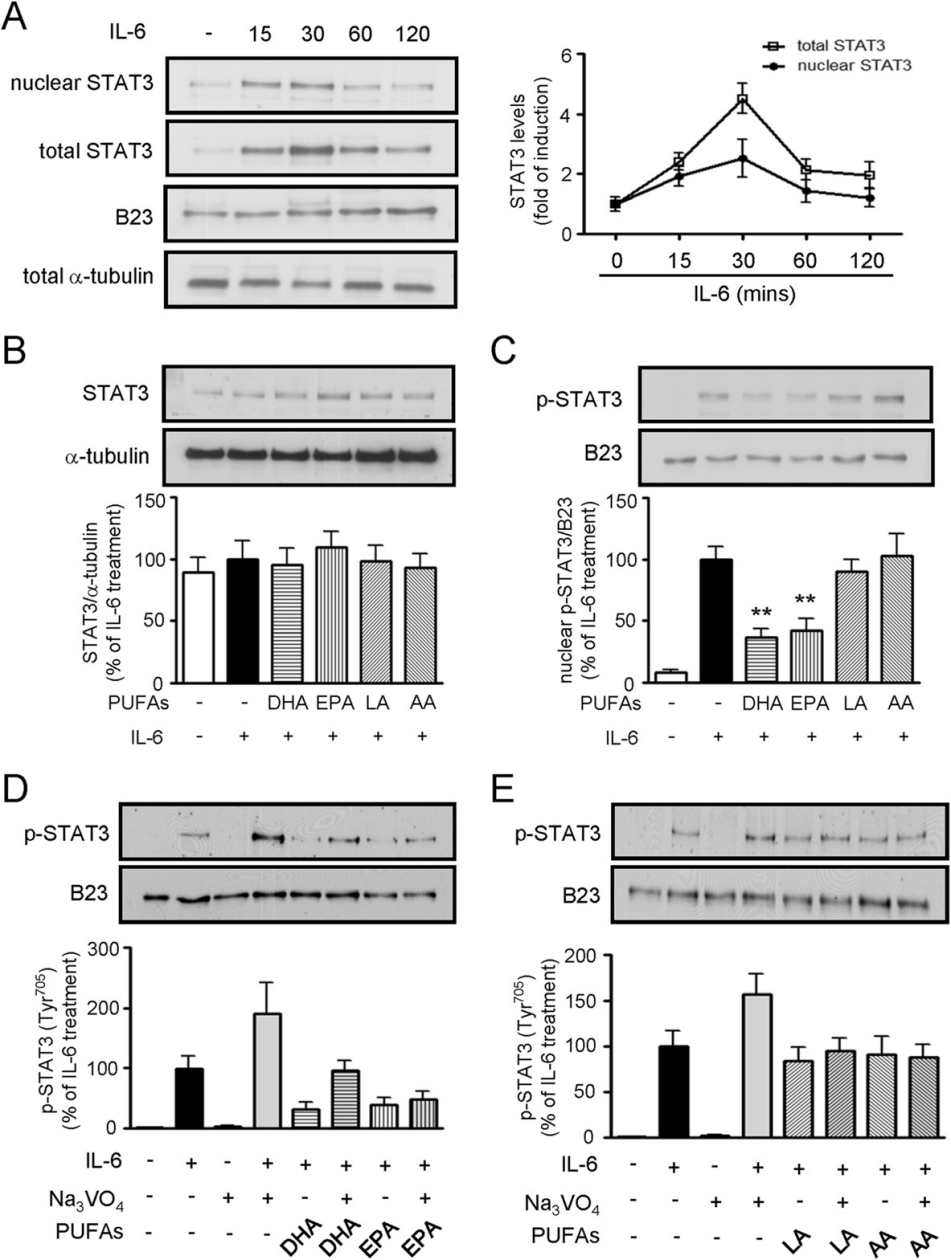
**Effects of PUFAs on IL-6-induced STAT3 signaling pathway. (A)** Time-dependent effect of IL-6 on STAT3 levels in the nucleus and total cell lysates was measured by western blot analysis. The expression of B23 and α-tubulin was used as the internal control for the nuclear protein and cytoplasmic protein, respectively. **(B)** Immunoblot analysis of whole cell lysates from cells pretreated with 100 μM PUFAs for 24 h and followed by stimulation with IL-6 for 30 min. **(C)** Parallel experiments as in panel B, but detecting STAT3 phosphorylation in the nucleus. Data were quantified by densitometry and the value of the IL-6-treated group was set as 100%. The results are presented as the mean ± SEM of at least three independent experiments, **P < 0.01 compared to the IL-6-treated group. **(D)** Cells were treated with IL-6 in absence or presence of the protein tyrosine phosphatase (PTPase) inhibitor Na_3_VO_4_ for 1 h. The effects of DHA and EPA on the phospho-Tyr^705^-STAT3 levels in the nucleus were determined by immunoblot analysis. **(E)** Parallel experiments similar to those described in panel D, but observing the effect of LA and AA. The photograph depicts a representative gel from three independent experiments.

Since IL-6 has been reported to enhance STAT3 expression [22], we then investigated the effect of PUFAs on STAT3 signaling in the IL6-stimulated HepG2 cells. As shown in Figure 5A, the time-course experiments revealed that total and nuclear STAT3 expression was activated to the greatest degree at 30 min of IL-6 treatment. We found that, although none of the PUFAs affected the level of STAT3 expression in whole cell lysates (Figure 5B), DHA and EPA displayed a significant suppressive effect on nuclear STAT3 phosphorylation at Tyr705 residue (Figure 5C). In contrast, LA and AA did not have any inhibitory effect on nuclear STAT3 phosphorylation. We further examined whether the suppressive effect of DHA and EPA on STAT3 phosphorylation might occur as a result of the reaction activity of a protein tyrosine phosphatase (PTPase) in HepG2 cells. We found that the broad-acting PTPase inhibitor sodium pervanadate (Na_3_VO_4_) was able to remarkably reverse the suppressive effect of DHA on STAT3 phosphorylation (Figure 5D), but did not affect the effect of EPA, LA, and AA (Figure 5D, 5E). These findings suggest that PTPase activity is involved in the mechanism associated with the suppressive effect of DHA on nuclear STAT3 expression.

## Discussion and conclusions

Dietary PUFAs are mostly delivered to the liver [23] and this organ is an essential metabolic center in animals and humans. The objective of this study was to investigate the impact of an elevated PUFA status on hepatic protein expression using an animal model and the regulatory mechanisms of CRP expression in PUFA-treated HepG2 cells. Using a proteomics approach, we pinpointed the PUFA-induced changes in the hepatic protein expression profile. Proteomics is a useful platform in nutrigenomics as it allows us to determine gene expression changes in response to diet [24,25]. Furthermore, proteomics represents a promising tool to uncover the mechanisms by which nutrients act and to identify the potential of food ingredients with respect to the maintenance of health or prevention of diseases [26,27]. This work is the first report to describe the proteome analysis of liver tissues from apoE-KO mice that have been treated with different PUFAs.

Our proteomic approach revealed 105 differentially expressed hepatic proteins in response to the four PUFAs. Further analysis of the PUFA-regulated proteins showed that most of the proteins involved in metabolism were up-regulated, though DHA and EPA induced a decrease in the expression of transcription factor sterol regulatory element-binding protein-1 (SREBP-1) and pyruvate carboxylase (PC). SREBP-1 has been proposed to be a lipogenic transcription factor central to lipogenesis and cholesterol metabolism [28] and PC has been shown to play a role in both glucose and lipid metabolism [29,30]. Thus, one consequence of the suppression of SREBP-1 and PC expression is a reduction in *de novo* lipogenesis and a disruption of glucose metabolism. These proteins and their pathways are potential therapeutic targets with respect to atherosclerosis and type 2 diabetes [31,32]. In addition, two enzymes (fructose-1,6-bisphosphatase 1 and alpha enolase), which are responsible for glucose metabolism, and three other enzymes (malate dehydrogenase, isocitrate dehydrogenase, ATP synthase), which are responsible for energy metabolism, are induced more than 2-fold in the n-3 PUFA-treated animals. Taken together, our proteomics data show that PUFAs may possibly alter fatty acid oxidation as well as energy, glucose, and amino acid metabolism.

In addition to the effect of PUFAs on lipid, carbohydrate, and energy metabolism, an increased intake of PUFAs may also lead to enhance oxidative stress [33] or protect organs against oxidative damage and inflammation [34]. Among the 105 identified hepatic proteins, 29 proteins were found to be involved in a number of biological functions associated with redox stress and inflammatory processes. Liver is a primary target organ to relieve oxidative stress, which is considered deleterious to the tissue [35]. From the findings of the proteomics analysis, it is clear that both DHA and EPA can remarkably enhance the hepatic expression of peroxiredoxin 6 (Prdx6), which is a cytoprotective protein that is capable of reducing the production of hydrogen peroxide [36]. Antioxidant enzymes such as superoxide dismutase 1 and glutathione peroxidase 1 are also induced by AA and LA, respectively, in the mouse liver. In addition, increased production of a number of glutathione S-transferases (GST), which are involved in antioxidant activity and the detoxification of carcinogens and drugs [37], were found to be induced in the liver of mice treated with various PUFAs. Furthermore, a remarkable increase, 6.9-fold compared with that of the control group, in the amount of selenium bind protein 2 (SBP2) was observed in the liver of EPA-treated mice. In contrast, SBP2 levels were suppressed in the DHA and AA-treated groups. These discrepancies may be the result of the diverse regulatory mechanisms through which different PUFAs act. Further research is needed to extend our knowledge of PUFA physiological functions.

The association of PUFAs with pro- or anti-inflammatory functions in liver cells is still not completely understood. Thus, we focused our studies on PUFAs that affected the expression of proteins related to inflammation. Unexpectedly, we found that 13 PUFA-modulated proteins are potentially involved in pro-inflammation, including annexin A2, interleukin 1 receptor accessory, MAP kinase kinase 3, A-kinase anchor protein, DC-SIGN related protein 1, G-protein coupled receptor (GPCR), interleukin 6 receptor-α, cyclophilin A, interferon regulatory factor, adrenoleukodystrophy protein, glutathione transferase ω-1, heat shock protein 60, and mitogen-activated protein kinase 10. These findings suggest that increased dietary PUFA supplementation may enhance inflammatory injury in the liver. A pro-inflammatory process is known to be a feature of the complex pro-atherogenic phenotype [38]. However, much of the interest in fatty acid nutrition has focused on the role of the n-3 fatty acids as anti-inflammatory protective agents [12,39], although little attention has yet been given to the possible role of n-3 fatty acids in the liver.

A close examination of the differentially expressed proteins from our proteomic analysis showed that a majority of PUFA-regulated proteins are inflammation related and associated with the NF-κB signaling pathway. Increasing evidence suggests that NF-κB is a key transcription factor that modulates the genes responsible for the immune response and inflammation [21,40]. Dysregulation of the NF-κB pathway plays an essential role in a number of diseases, including inflammatory diseases, cancer, and atherosclerosis [41,42]. Based on these, we extended our investigation to elucidate whether PUFA treatment affected the NF-κB-signaling pathway in HepG2 cells.

IL-1 and IL-6 are the two potent inflammation-associated cytokines that play essential roles in the regulation of immune and inflammatory reactions [43,44] and can also act as stimulators of CRP production [45,46]. CRP is synthesized and secreted mainly by the liver in response to circulating inflammatory mediators [47]. Elevated CRP levels also serve as a risk marker for cardiovascular disease, cancer, and mortality [48,49]. Although various roles for CRP in different diseases have been suggested, little information is available regarding whether this protein expression is regulated by PUFAs in the liver. Thus, we identified possible signaling pathways that are triggered by cytokines and investigated whether CRP gene expression was altered by PUFA treatment in HepG2 cells.

**Figure 6:**
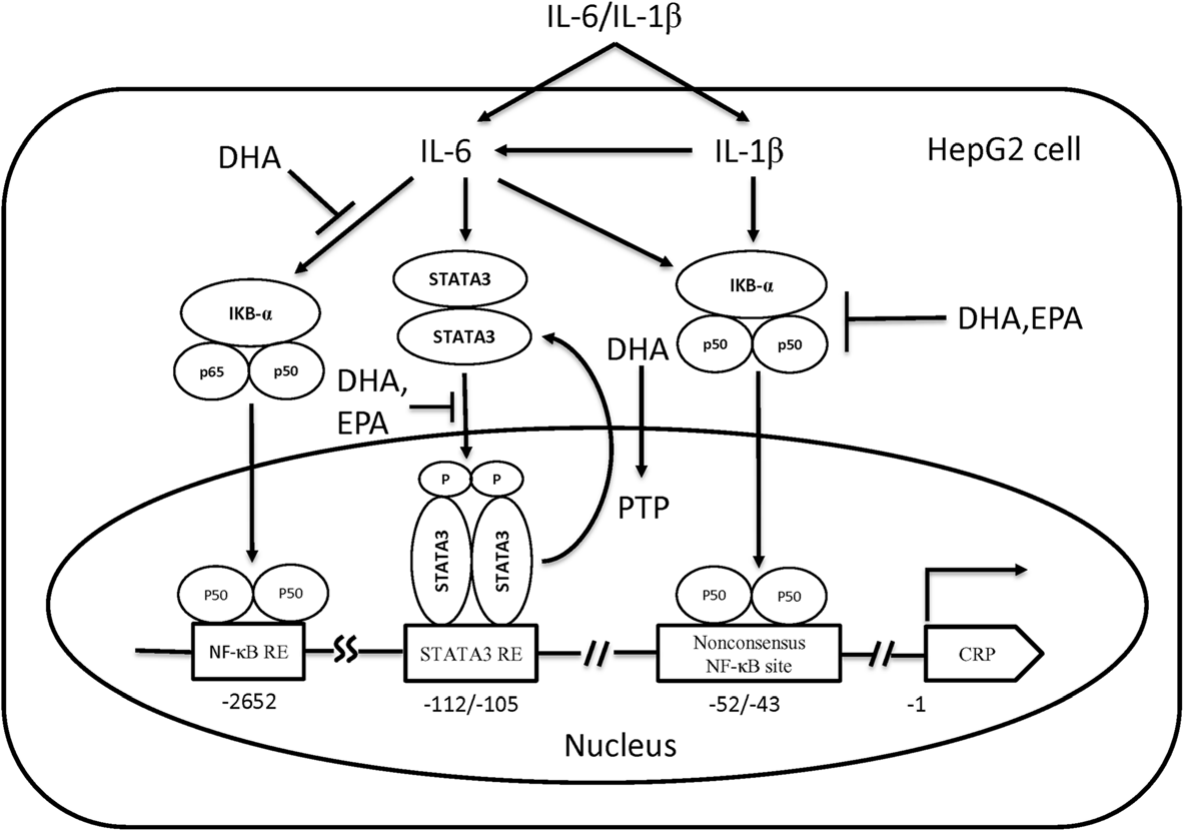
**Schematic representation of the mechanisms in the regulation of** ***CRP*** **gene expression by n-3 PUFAs in the IL1β/IL6-challenged HepG2 cells.**

To our knowledge, this study is the first to offer insights into the global regulation of hepatic protein expression in PUFA-treated apoE-KO mice. In the present study, we elucidate how PUFAs exert their influence on hepatic metabolism, redox stress/inflammation, enzymatic reactions, and miscellaneous functions. We propose that n-3 PUFAs suppress the cytokine-activated NF-κB signaling, which would then result in the subsequent regulation of *CRP* gene expression in liver cells. Our data show that DHA suppresses the IL-6-induced translocation of nuclear NF-κB subunits p50/p65 heterodimer and p50/p50 homodimer. In contrast, EPA only inhibits the expression of p50/p50. Furthermore, EPA is able to inhibit STAT3 activation through suppression of STAT3 phosphorylation, while DHA suppresses STAT3 activation both through suppression of STAT3 phosphorylation and induction of PTPase activity. PTPases have been known to play as the negative regulators in STAT3 activation [50]. STAT3 is an acute-phase response factor activated by phosphorylation at Tyr705, such activation leads to IL-6 regulation of many acute-phase protein genes [51]. Although Janus kinases (JAKs) pathway may also play as the negative regulator by cytokine-induce inflammation in different diseases [52], we mainly focus on the investigation of STAT3 signaling because that STAT3 is the main mediator of IL-6 induction in the liver [53]. In the present study, our data show that DHA and EPA suppress the IL6-induced Tyr705 phosphorylation of STAT3, which in turn inhibit the transcription activation of *CPR* gene. In the diagram presented in Figure 6, we suggest the differential regulatory mechanisms whereby DHA and EPA suppress IL-1/IL-6-induced CRP gene expression.

## Abbreviations

AA: Arachidonic acid
ApoE-KO: ApoE-knockout
CRP: C-reactive protein
DHA: Docosahexaenoic acid
EPA: Eicosapentaenoic acid
HDL-C: High-density lipoprotein-cholesterol
IL: Interleukin
LA: Linoleic acid
LDL-C: Low-density lipoprotein-cholesterol
MTT: 3-(4,5-dimethylthiazol-2-yl)-2,5-diphenyltetrazolium bromide
NF-κB: Nuclear factor-κB
PC: Pyruvate carboxylase
PUFAs: Polyunsaturated fatty acids
PMSF: Phenylmethanesulfonyl fluoride
STAT3: Signal transducer and activator of transcription 3
SREBP-1: Sterol regulatory element-binding protein-1
TC: Total cholesterol
TG: Triacylglycerol

## References

[1] Song Z, Yang L, Shu G, Lu H, Sun G (2013). Effects of the n-6/n-3 polyunsaturated fatty acids ratio on postprandial metabolism in hypertriacylglycerolemia patients. Lipids Health Dis.

[2] Janssen CI, Kiliaan AJ (2014). Long-chain polyunsaturated fatty acids (LCPUFA) from genesis to senescence: the influence of LCPUFA on neural development, aging, and neurodegeneration. Prog Lipid Res.

[3] Lombardo YB, Chicco AG (2006). Effects of dietary polyunsaturated n-3 fatty acids on dyslipidemia and insulin resistance in rodents and humans. A review. J Nutr Biochem.

[4] Im DS (2012). Omega-3 fatty acids in anti-inflammation (pro-resolution) and GPCRs. Prog Lipid Res.

[5] Wang TM, Chen CJ, Lee TS, Chao HY, Wu WH, Hsieh SC (2011). Docosahexaenoic acid attenuates VCAM-1 expression and NF-kappaB activation in TNF-alpha-treated human aortic endothelial cells. J Nutr Biochem.

[6] de Roos B, Mavrommatis Y, Brouwer IA (2009). Long-chain n-3 polyunsaturated fatty acids: new insights into mechanisms relating to inflammation and coronary heart disease. Br J Pharmacol.

[7] Aarsetoey H, Grundt H, Nygaard O, Nilsen DW (2012). The role of long-chained marine N-3 polyunsaturated fatty acids in cardiovascular disease. Cardiol Res Pract.

[8] Weber C, Noels H (2011). Atherosclerosis: current pathogenesis and therapeutic options. Nat Med.

[9] Moore KJ, Tabas I (2011). Macrophages in the pathogenesis of atherosclerosis. Cell.

[10] Wiernsperger N (2013). Hepatic function and the cardiometabolic syndrome. Diabetes Metab Syndr Obes.

[11] Ludwig T, Worsch S, Heikenwalder M, Daniel H, Hauner H, Bader BL (2013). Metabolic and immunomodulatory effects of n-3 fatty acids are different in mesenteric and epididymal adipose tissue of diet-induced obese mice. Am J Physiol Endocrinol Metab.

[12] Yates CM, Calder PC, Ed Rainger G (2014). Pharmacology and therapeutics of omega-3 polyunsaturated fatty acids in chronic inflammatory disease. Pharmacol Ther.

[13] Spiller OB, Criado-Garcia O, Rodriguez De Cordoba S, Morgan BP (2000). Cytokine-mediated up-regulation of CD55 and CD59 protects human hepatoma cells from complement attack. Clin Exp Immunol.

[14] Stapp JM, Sjoelund V, Lassiter HA, Feldhoff RC, Feldhoff PW (2005). Recombinant rat IL-1beta and IL-6 synergistically enhance C3 mRNA levels and complement component C3 secretion by H-35 rat hepatoma cells. Cytokine.

[15] Szabo G, Csak T (2012). Inflammasomes in liver diseases. J Hepatol.

[16] Black S, Kushner I, Samols D (2004). C-reactive Protein. J Biol Chem.

[17] Tyakht AV, Ilina EN, Alexeev DG, Ischenko DS, Gorbachev AY, Semashko TA (2014). RNA-Seq gene expression profiling of HepG2 cells: the influence of experimental factors and comparison with liver tissue. BMC Genomics.

[18] Folch J, Lees M, Sloane Stanley GH (1957). A simple method for the isolation and purification of total lipides from animal tissues. J Biol Chem.

[19] Liao KA, Tsay YG, Huang LC, Huang HY, Li CF, Wu TF (2011). Search for the tumor-associated proteins of oral squamous cell carcinoma collected in Taiwan using proteomics strategy. J Proteome Res.

[20] Patel DN, King CA, Bailey SR, Holt JW, Venkatachalam K, Agrawal A (2007). Interleukin-17 stimulates C-reactive protein expression in hepatocytes and smooth muscle cells via p38 MAPK and ERK1/2-dependent NF-κB and C/EBPβ activation. J Biol Chem.

[21] Ghosh S, Hayden MS (2008). New regulators of NF-kappaB in inflammation. Nat Rev Immunol.

[22] Zhang D, Sun M, Samols D, Kushner I (1996). STAT3 participates in transcriptional activation of the C-reactive protein gene by interleukin-6. J Biol Chem.

[23] Donnelly KL, Smith CI, Schwarzenberg SJ, Jessurun J, Boldt MD, Parks EJ (2005). Sources of fatty acids stored in liver and secreted via lipoproteins in patients with nonalcoholic fatty liver disease. J Clin Invest.

[24] Dang TS, Walker M, Ford D, Valentine RA (2014). Nutrigenomics: the role of nutrients in gene expression. Periodontol.

[25] Ordovas JM, Corella D (2004). Nutritional genomics. Annu Rev Genomics Hum Genet.

[26] Low YL, Tai ES (2007). Understanding diet-gene interactions: lessons from studying nutrigenomics and cardiovascular disease. Mutat Res.

[27] de Roos B, McArdle HJ (2008). Proteomics as a tool for the modelling of biological processes and biomarker development in nutrition research. Br J Nutr.

[28] Karasawa T, Takahashi A, Saito R, Sekiya M, Igarashi M, Iwasaki H (2011). Sterol regulatory element-binding protein-1 determines plasma remnant lipoproteins and accelerates atherosclerosis in low-density lipoprotein receptor-deficient mice. Arterioscler Thromb Vasc Biol.

[29] Vessal M, Mishra S, Moulik S, Murphy LJ (2006). Prohibitin attenuates insulin-stimulated glucose and fatty acid oxidation in adipose tissue by inhibition of pyruvate carboxylase. FEBS J.

[30] MacDonald MJ, Hasan NM, Dobrzyn A, Stoker SW, Ntambi JM, Liu X (2013). Knockdown of pyruvate carboxylase or fatty acid synthase lowers numerous lipids and glucose-stimulated insulin release in insulinoma cells. Arch Biochem Biophys.

[31] Han J, Liu YQ (2010). Reduction of islet pyruvate carboxylase activity might be related to the development of type 2 diabetes mellitus in Agouti-K mice. J Endocrinol.

[32] Li Y, Xu S, Jiang B, Cohen RA, Zang M (2013). Activation of sterol regulatory element binding protein and NLRP3 inflammasome in atherosclerotic lesion development in diabetic pigs. PLoS One.

[33] Kello M, Mikes J, Jendzelovsky R, Koval J, Fedorocko P (2010). PUFAs enhance oxidative stress and apoptosis in tumour cells exposed to hypericin-mediated PDT. Photochem Photobiol Sci.

[34] Castillo RL, Arias C, Farias JG (2014). Omega 3 chronic supplementation attenuates myocardial ischaemia-reperfusion injury through reinforcement of antioxidant defense system in rats. Cell Biochem Funct.

[35] McMillian M, Nie A, Parker JB, Leone A, Kemmerer M, Bryant S (2005). Drug-induced oxidative stress in rat liver from a toxicogenomics perspective. Toxicol Appl Pharmacol.

[36] Flohe L, Budde H, Hofmann B (2003). Peroxiredoxins in antioxidant defense and redox regulation. Biofactors.

[37] Hayes JD, Pulford DJ (1995). The glutathione S-transferase supergene family: regulation of GST and the contribution of the isoenzymes to cancer chemoprotection and drug resistance. Crit Rev Biochem Mol Biol.

[38] Margioris AN (2009). Fatty acids and postprandial inflammation. Curr Opin Clin Nutr Metab Care.

[39] Wall R, Ross RP, Fitzgerald GF, Stanton C (2010). Fatty acids from fish: the anti-inflammatory potential of long-chain omega-3 fatty acids. Nutr Rev.

[40] Sun SC (2012). The noncanonical NF-kappaB pathway. Immunol Rev.

[41] Pikarsky E, Porat RM, Stein I, Abramovitch R, Amit S, Kasem S (2004). NF-kappaB functions as a tumour promoter in inflammation-associated cancer. Nature.

[42] de Winther MP, Kanters E, Kraal G, Hofker MH (2005). Nuclear factor kappaB signaling in atherogenesis. Arterioscler Thromb Vasc Biol.

[43] Kamimura D, Ishihara K, Hirano T (2003). IL-6 signal transduction and its physiological roles: the signal orchestration model. Rev Physiol Biochem Pharmacol.

[44] Dinarello CA (2011). A clinical perspective of IL-1beta as the gatekeeper of inflammation. Eur J Immunol.

[45] Zhang D, Jiang SL, Rzewnicki D, Samols D, Kushner I (1995). The effect of interleukin-1 on C-reactive protein expression in Hep3B cells is exerted at the transcriptional level. Biochem J.

[46] Li SP, Goldman ND (1996). Regulation of human C-reactive protein gene expression by two synergistic IL-6 responsive elements. Biochemistry.

[47] Du Clos TW, Mold C (2004). C-reactive protein: an activator of innate immunity and a modulator of adaptive immunity. Immunol Res.

[48] Venugopal SK, Devaraj S, Jialal I (2005). Effect of C-reactive protein on vascular cells: evidence for a proinflammatory, proatherogenic role. Curr Opin Nephrol Hypertens.

[49] Allin KH, Nordestgaard BG (2011). Elevated C-reactive protein in the diagnosis, prognosis, and cause of cancer. Crit Rev Clin Lab Sci.

[50] Lee JH, Chiang SY, Nam D, Chung WS, Lee J, Na YS (2014). Capillarisin inhibits constitutive and inducible STAT3 activation through induction of SHP-1 and SHP-2 tyrosine phosphatases. Cancer Lett.

[51] Heinrich PC, Behrmann I, Haan S, Hermanns HM, Müller-Newen G, Schaper F (2003). Principles of interleukin (IL)-6-type cytokine signalling and its regulation. Biochem J.

[52] Jenkins BJ (2014). Transcriptional regulation of pattern recognition receptors by Jak/STAT signaling, and the implications for disease pathogenesis. J Interferon Cytokine Res.

[53] Alonzi T, Maritano D, Gorgoni B, Rizzuto G, Libert C, Poli V (2001). Essential role of STAT3 in the control of the acute-phase response as revealed by inducible gene activation in the liver. Mol Cell Biol.

